# How the love of muscle can break a heart: Impact of anabolic androgenic steroids on skeletal muscle hypertrophy, metabolic and cardiovascular health

**DOI:** 10.1007/s11154-020-09616-y

**Published:** 2020-12-02

**Authors:** Deaglan McCullough, Richard Webb, Kevin J. Enright, Katie E. Lane, Jim McVeigh, Claire E. Stewart, Ian G. Davies

**Affiliations:** 1 Research Institute of Sport and Exercise Science, Liverpool John Moores University, Liverpool, UK; 2 Faculty of Science, Liverpool Hope University, Liverpool, UK; 3 Substance Use and Associated Behaviours Group, Manchester Metropolitan University, Manchester, UK

**Keywords:** Anabolic-androgenic steroids, Metabolic syndrome, High-density lipoprotein cholesterol, Low-density lipoprotein cholesterol, Insulin resistance, Cardiovascular disease

## Abstract

It is estimated 6.4% of males and 1.6% of females globally use anabolic-androgenic steroids (AAS), mostly for appearance and performance enhancing reasons. In combination with resistance exercise, AAS use increases muscle protein synthesis resulting in skeletal muscle hypertrophy and increased performance. Primarily through binding to the androgen receptor, AAS exert their hypertrophic effects via genomic, non-genomic and anti-catabolic mechanisms. However, chronic AAS use also has a detrimental effect on metabolism ultimately increasing the risk of cardiovascular disease (CVD). Much research has focused on AAS effects on blood lipids and lipoproteins, with abnormal concentrations of these associated with insulin resistance, hypertension and increased visceral adipose tissue (VAT). This clustering of interconnected abnormalities is often referred as metabolic syndrome (MetS). Therefore, the aim of this review is to explore the impact of AAS use on mechanisms of muscle hypertrophy and markers of MetS. AAS use markedly decreases high-density lipoprotein cholesterol (HDL-C) and increases low-density lipoprotein cholesterol (LDL-C). Chronic AAS use also appears to cause higher fasting insulin levels and impaired glucose tolerance and possibly higher levels of VAT; however, research is currently lacking on the effects of AAS use on glucose metabolism. While cessation of AAS use can restore normal lipid levels, it may lead to withdrawal symptoms such as depression and hypogonadism that can increase CVD risk. Research is currently lacking on effective treatments for withdrawal symptoms and further long-term research is warranted on the effects of AAS use on metabolic health in males and females.

## Introduction

The fine margins of winning and losing in athletic competitions has always encouraged innovative techniques to help athletes gain a competitive advantage with little regard to the potential negative consequences. Although research into sex hormones existed in the early 19th century, it was only in the 1930s when the anabolic effects of testosterone were demonstrated [1]. Shortly thereafter, the hormone started to be used by competitive athletes to increase muscle mass and performance, however, the British Association of Sports Medicine and the American College of Sports Medicine continued to deny its potential benefits until the 1970s [2, 3]. The use of testosterone and its derivatives were later banned by the International Olympics Committee in 1974 [4]. Due to advancements in technology and pharmacology, a range of anabolic androgenic steroids (AAS) (Table 1, [5, 6]) began to be commonly used by the recreational gym-user in the 1980s, primarily by young men to improve body image [1, 7]. Due to this rise in use and the associated adverse effects of AAS, many countries changed their legislation to incorporate AAS to regulate its use and distribution in the 1990s [8–10]. The world anti-doping agency was created in 1999 to protect athletes from the detrimental health risks of AAS use and to ensure maintenance of the integrity of sport globally [4, 11].

It is currently estimated that 6.4% of males and 1.6% of females use AAS globally, with recreational sportspeople being the highest users [12]. Although it is common for individuals to use AAS for multiple reasons, the greatest motivation to use AAS is primarily to improve body image, while competitive bodybuilding and athletic performance (non-bodybuilding) are secondary and tertiary respectively [12–15]. The Middle East has relatively significantly high levels of AAS use while use in South America, Europe, North America, Oceania and Africa ranges from 5–2% of the population, highlighting the global issue at hand [12]. However, the significantly higher prevalence rates in the Middle East may be due to the majority of studies relying on self-reports from athletes rather than general populations [12]

**Table 1:** List of injectable and oral AAS and typical doses used

Injectable AAS	Typical weekly dose
Boldenone Undecanoate	200–400 mg
Drostanolone Propionate	300–450 mg
Methenolone Enanthate	200–400 mg
Nandrolone Decanoate	200–400 mg
Stanozolol	150–700 mg
Testosterone Cypionate	200–600 mg
Testosterone Enanthate	200–600 mg
Testosterone Propionate	150–300 mg
Testosterone Suspension	150–700 mg
Trenbolone Acetate	150–300 mg
Trenbolone Enanthate	200–300 mg
Trenbolone Hexahydrobenzylcarbonate	150–230 mg
Oral AAS	Daily dose
4-chlorodehydromethyltestosterone	20–80 mg
Fluoxymesterone	20–40 mg
Methandrostenolone, Methandienone	20–40 mg
Mesterolone	50–100 mg
Oxandrolone	20–40 mg
Oxymetholone	50–100 mg
Stanozolol	20–50 mg
Testosterone Undecanoate	80–160 mg

### Effects on skeletal muscle

**Fig. 1:**
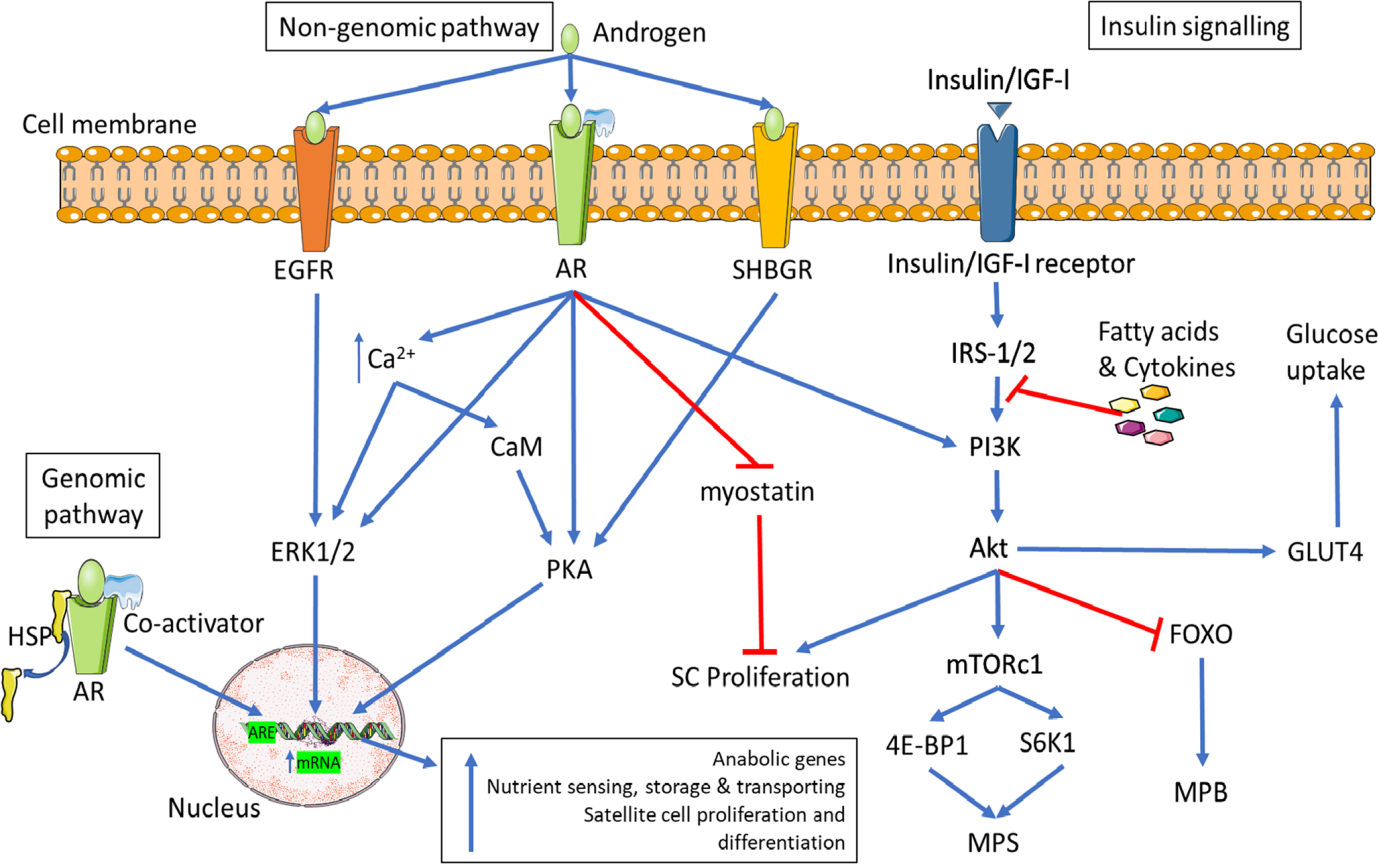
Genomic and non-genomic mechanisms of AAS induced skeletal muscle hypertrophy and mechanisms of insulin signalling and resistance. Genomic pathway: Androgen binding of the AR complex causes translocation to the nucleus following dissociation of heat shock proteins (HSP). The androgen/AR complex regulates gene transcription on the androgen response element (ARE) of DNA. Non-genomic pathway: In addition to the AR, androgens can activate other membrane-bound receptors such as EGFR and SHBGR. This causes an increase in intracellular calcium (Ca2+), activation of several second messenger signalling such as extracellular regulated kinases 1/2 (ERK 1/2), protein kinase A (PKA), calmodulin (CaM) and phosphatidylinositol-3-phosphate kinase (PI3K)/Akt/mTORc1 pathways and deactivation of myostatin pathway. Activation of these genomic and non-genomic pathways leads to skeletal muscle hypertrophy via upregulating gene transcription of anabolic genes, nutrient sensing, storage and transporting. While also upregulating satellite cell proliferation, differentiation, MPS and inhibiting muscle protein breakdown (MPB). Insulin/IGF-1 signalling pathway: Insulin/IGF-1 bind to the insulin/IGF-1 receptor on the cell membrane inflicting tyrosine phosphorylation. The now activated receptor causes phosphorylation of insulin receptor substrate-1/2 (IRS-1/2) activating the PI3K/Akt signalling cascade leading to satellite cell proliferation; MPS via mTORc1, 4E-binding protein 1 (4E BP1) and p70 S6 kinase 1 (S6K1) activation; glucose uptake via GLUT4 translocation and inhibition of forkhead O transcription factor (FOXO) leading to reduced MPB. Abnormal levels of circulating fatty acids and inflammatory cytokines result in serine/threonine phosphorylation of IRS-1 causing insulin resistance.

Testosterone and its AAS derivatives increase muscle protein synthesis (MPS) and accretion, satellite cell activation and possibly decrease catabolic pathways via genomic and non-genomic mechanisms (Fig. 1) [16]. Genomic actions of AAS occur when androgens bind to the nuclear androgen receptor (AR) and translocate to the cell nucleus, binding to specific DNA sequences resulting in enhanced transcription of target anabolic genes [17, 18]. AAS also exert non-genomic actions by binding of the membrane-located AR and additional membrane receptors such as endothelial growth factor receptor (EGFR) and sex hormone-binding globulin receptor (SHBGR) that also alter anabolic/catabolic signalling pathways [17, 18]. Resistance exercise also increases muscle MPS and satellite cell activation resulting in skeletal muscle hypertrophy [19, 20]. Although testosterone administration and resistance exercise alone may increase skeletal muscle hypertrophy, the combination of both results in enhanced skeletal muscle hypertrophy [21]. As a result, AAS are commonly used in conjunction with exercise to increase muscle mass and improve perceived body image [1, 23].

### Effects on metabolic health

Regular exercise is undoubtedly beneficial for mental, physical and metabolic health [22]. However, the potential benefits acquired from regular exercise may be reduced with chronic AAS use as AAS users are at a higher risk of developing cardiovascular disease (CVD), psychological disorders, neuroendocrine disorders, sex-specific disorders (aromatisation and hypogonadism in males and virilisation in females) and a range of other disorders (Table 2) [7, 23–26]. Long term AAS use has been shown to result in premature death due to cardiovascular events; however, due to AAS use only being prevalent since the 1980s, long term longitudinal studies, on their impact, are scarce [27]. Furthermore, the direct impact of AAS use on health is difficult to determine as users reportedly use other substances to complement their AAS use while also using a variety of AAS types, doses and cycles [13, 28]. AAS-related polysubstance use also includes other anabolic agents such as insulin-like growth factor-I (IGF-I) and growth hormone (hGH); drugs to prevent AAS-related adverse effects, other image enhancing drugs (clenbuterol, diuretics and thyroid hormones) and psychoactive drugs [13, 28]. The chemical interactions of AAS-related polysubstance use may also elicit additional adverse health outcomes. Quantifying the adverse effects of these drugs is further complicated by the prevalence of adulterated products, an inevitable consequence of the illicit market [29].

Metabolic syndrome (MetS) is the constellation of the often interrelated metabolic abnormalities that lead to increased risk of CVD, which are the number one cause of death globally [30, 31]. It is most commonly associated with sedentary/obese populations and is defined by having a combination of some, but not all, of high triglycerides (TG), low high-density lipoprotein cholesterol (HDL-C), elevated blood glucose, hypertension and elevated waist circumference [30, 32]. Insulin resistance (IR), visceral adipose tissue (VAT) and small dense low-density lipoprotein cholesterol (sdLDL-C) also highly correlate with MetS [30]. Although AAS users are highly active, they are also at risk of CVD as AAS use has been reported to increase the risk of sudden cardiac arrest as a result of cardiac remodelling and abnormal cardiac function [33–35]. The use of AAS reportedly results in polycythaemia, reduced left ventricular and diastolic function and accelerated atherosclerosis compared to non-use [24, 36]. AAS use may affect blood pressure (BP) and metabolism which ultimately increases CVD risk in addition to altered cardiac function [33]. Furthermore, AAS use can increase low-density lipoprotein cholesterol (LDL-C) and decrease high-density lipoprotein cholesterol (HDL-C) increasing the risk of developing atherosclerosis and hence CVD [33], particularly given that AAS use could result in lower insulin sensitivity and higher levels of VAT compared to matched controls [37]. Therefore, although most AAS users have high levels of activity and low adiposity, they can also share similar metabolic characteristics of obese/sedentary populations such as the MetS, thereby increasing risk of CVD.

**Table 2:** Diseases associated with chronic AAS use

Cardiovascular	Psychological	Neuroendocrine	Other
Cardiomyopathy	Depression	Neurotoxicity	Hepatoxicity
Coronary heart disease	Mood disorders	Reduced grey matter	Hypogonadism (males)
Sudden cardiac death	Substance abuse	Thinner and smaller cortices	Virilisation (females)
Stroke	Dependence	Cognitive impairment	Acne
Myocardial infarction			Fertility
Hypertension			Cancer

### AAS withdrawal

While increasing levels of lean mass has an inverse relationship with CVD risk, AAS use has such a deleterious effect on health that it is not recommended to use for appearance or performance reasons [7, 38]. Discontinuing AAS use can be a difficult process as immediate cessation may also have a detrimental effect on health and wellbeing. The withdrawal effects of AAS can cause hypogonadism, depression and fatigue, reduced libido, leading to relapse and AAS dependency [7, 39]. Current evidence on successful treatments for cessation of AAS use are scarce and further research is required, but potential strategies for males include testosterone replacement therapy (TRT), selective estrogen receptor modulators (SERM), human chorionic gonadotropin (hCG) and aromatase inhibitors [40, 41]. As a result, up to date guidance and information on the risks of commencing and ceasing AAS use along with effective treatments for withdrawal symptoms are required to prevent adverse health outcomes.

## Aim and scope

Although previous reviews have focused on the effects of AAS use on blood lipid and lipoproteins profiles [33, 42], the effects on overall metabolism have yet to be reviewed. Abnormal lipid metabolism is commonly associated with impaired glucose metabolism, hypertension and VAT accumulation and this may also be the case in AAS users [30]. Therefore, the objectives of this review are to: 1, highlight the mechanisms by which AAS exert their hypertrophic effects on skeletal muscle; 2, explore the impact of AAS use on lipid, lipoprotein and glucose metabolism, all indicators of MetS and 3, explore the negative effects of AAS withdrawal and potential treatments. With the substantial levels of AAS use [7, 12], better knowledge of these interrelated mechanisms and issues may lead to targeted interventions to reduce the potential harm that may be associated with AAS use.

## Mechanism of action on skeletal muscle

### Genomic-mediated mechanisms

The primary action of AAS is to bind to the nuclear AR located in the cytoplasm which results in their translocation to the nucleus following disassociation of the AR complex with chaperone (Hsp90, Hsp70) and co-chaperone proteins (Hsp organising protein (Hop)) [43, 44]. At the nucleus the androgen/AR complex moderates gene transcription by binding to the ARE of the DNA [17, 45]. Transcription is altered further by the recruitment co-activators such as cAMP response element-binding protein (CREB)-binding protein (CBP)/p300 and steroid receptor coactivator (Src) 1, 2 and 3 [17, 45, 46]. This, in turn, upregulates expression of genes related to protein accretion and anabolism such as IGF-I, nutrient sensing, storage and transporting (Lipin, GLUT3 and SAT2) and satellite cell differentiation (myogenin), while also increasing satellite cell number [47–50]. ARE binding may also downregulate genes involved in muscle atrophy such as I-Kappa kinase alpha (IKKα) [47, 51] (Fig. 1). The transactivation domain of the AR is susceptible to a CAG repeat polymorphism within the first exon, which may regulate AR activity. The number of CAG repeats typically ranges from 11 to 31 triplets in length and is inversely associated with transactivational activity of the AR [43]. An increase in CAG repeats is associated with elevated testosterone levels, perhaps due to decreased AR activity, which may affect hypothalamic-pituitary feedback regulation although no association has been observed with muscle mass in young males (25–45 years) [52]. There is currently a lack of evidence on the response of AAS in relation to the amount of AR CAG repeats.

### Non-genomic mediated adaptations

Non-genomic actions of AAS are characterised by the speed in which they exert their effects (within minutes) thus indicating activities independent of transcription [17, 53]. AAS have been reported to exert non-genomic effects via membrane-located receptors; membrane-located AR, EGFR, and SHBGR [17]. Binding of these receptors leads to an increase in intracellular calcium and activation of several second messenger signalling cascades including; mitogen-activated protein kinases (MAPK), ERK 1/2, PKA, PI3K/Akt and CaM pathways [17, 54, 55]. Activation of PI3K/Akt by testosterone, triggers mTORc1, a key regulator of protein turnover via activation of the eukaryotic initiation factor 4E-BPs and S6K1 [19, 56]. Resistance exercise also activates S6K1 via mTORc1, increasing MPS and muscle hypertrophy [57, 58]. The combination of testosterone and resistance exercise further increases mTORc1, 4E-BP1 and S6K1 activation compared to either alone [59]. These signalling cascades upregulate transcription, satellite cell proliferation, muscle protein synthesis and reduce apoptosis ultimately resulting in skeletal muscle hypertrophy [17, 54, 55, 60] (Fig. 1).

### Anti-catabolic effects of AAS

In addition to the genomic and non-genomic effects of AAS decreasing atrophy related gene expression and activity of catabolic pathways (FOXO pathway), AAS may cause direct inhibition on glucocorticoid receptor (GR) signalling and/or its expression [5]. Binding of the GR by agonists increases skeletal muscle atrophy and thereby inhibition of this pathway will increase net protein balance and further increase muscle hypertrophy [61]. Compared to young muscle, aged muscle shows a decrease in function and size, of which the mechanisms are multifactorial [62]. Increases in apoptosis may be implicated in this decline of ageing muscle as old mice were observed to have increased rates of apoptosis of skeletal muscle compared to young [63]. Treatment of old mice with testosterone reduced apoptotic rate to similar levels of young mice while also regenerating myofiber size [63]. Myostatin, an endogenous inhibitor of muscle growth through negative regulation of satellite cell proliferation and differentiation, was also shown to be downregulated following testosterone treatment in old mice, further highlighting its potential anti-catabolic effect [63, 64] (Fig. 1). However, the specific anti-catabolic effects of AAS use may only be beneficial in populations with abnormally low levels of testosterone such as ageing, as it remains to be confirmed in healthy adults with normal testosterone level.

### Resulting effect on muscle mass and/or performance

**Fig. 2:**
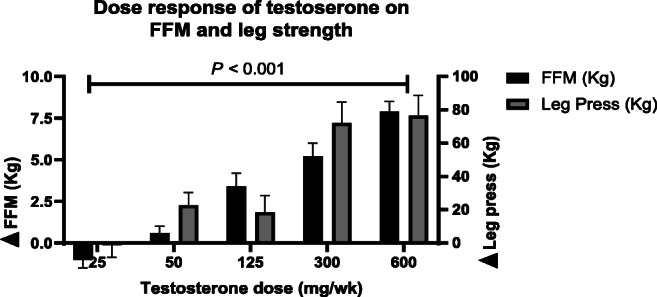
The dose-response effect of testosterone on change in fat-free mass (FFM) and leg press strength after 20 weeks in combination with a resistance exercise protocol (redrawn from Bhasin et al.) [65]

The enhanced anabolic and reduced catabolic signalling highlights the benefits of AAS use for enhanced muscle growth yet, it wasn’t until the mid-90 s that it was confirmed to result in improved athletic performance [21]. In 1996, Bhasin et al.. performed the first controlled experiment on the effects of testosterone enanthate (TestE) on muscle mass and strength in males (N = 40, 19–40 years) [21]. Energy and protein intake were match controlled and participants were randomly assigned to one of four groups (no exercise with or without TestE and exercise with or without TestE). Similar supervised training programmes were followed, and TestE groups injected 600 mg per week of TestE intramuscularly for 10 weeks. Muscle thickness and strength showed significant (*P <* 0.05) improvements in the TestE alone and exercise alone; however, the combination of TestE and exercise had a significant (*P <* 0.001) additive effect [21]. The enhanced effect of combining AAS and resistance exercise on muscle hypertrophy and strength is most likely due to upregulation of AR activation and enhanced mTORc1 signalling as discussed above [56]. It was later reported that there is a dose-response relationship (25–600 mg p/wk) of AAS with body composition and muscle performance (Fig. 2) [65]. Furthermore, supraphysiological doses of AAS (200–300 mg p/wk) significantly increased cycle performance compared to placebo-controlled participants following a 6-week resistance exercise programme [66]. Self-reported AAS use suggests that the enhanced effects on muscle mass and strength continue to be elevated with long term use (> 5 years) compared to non-users [67].

In physically active females (mean age 24 years old), 10 weeks of testosterone cream treatment (70 mg/wk) resulted in a significant (*P* < 0.001) moderate increase in testosterone levels (0.9 to 4.3 nmol/L). This increase in testosterone levels resulted in significantly (*P* < 0.05) improved lean mass compared to a placebo-controlled group but no improvement in muscle strength was detected [68]. Although 10 weeks may not be long enough to increase muscle strength, higher doses of AAS (up to 600 mg per week) may show greater improvements due to the dose-response relationship of AAS and muscular improvements, however, data are limited in females [65]. Furthermore, female athletes with higher endogenous testosterone levels have an increase in athletic performance in 400 m, 400 m hurdles, 800 m, hammer throw and pole vault by 2–5% [69]. A recent systematic review and meta-analysis on the effects of AAS on healthy exercising adults of all ages showed that AAS use with exercise improved strength by 52% along with improved body composition vs. non-users [70]. Although data reported were highly variable, ranging in quality and lacking female representation, the evidence of translating the enhanced anabolic signalling into increased muscle mass and performance is substantial. Due to this, AAS have been viewed as a possible strategy in reducing the age-related decline in muscle mass and function (termed sarcopenia) in testosterone deficient older individuals [71]. Although resistance exercise has shown to reduce the severity of sarcopenia [72, 73], serum testosterone levels decline with age in males which may lead to testosterone deficiency and attribute to sarcopenia and frailty; therefore, low dose testosterone supplementation may attenuate this decline and even improve muscle mass and function [74]. Long term (> 3 years) TRT (~ 75 mg daily to achieve normal total testosterone levels) in older (> 60 years) men resulted in improvements in muscle strength, power and lean body mass compared to a placebo-controlled group [75]. Additionally, the testosterone trials involved 7 coordinated placebo-controlled trials with the aim of increasing deficient testosterone levels to normal levels in 788 males aged ≥ 65 years old with transdermal TRT for one year [76]. TRT resulted in improving 6-minute walking distance in addition to increasing sexual function, mood and bone mineral density [77].

Nonetheless, TRT is only recommended for individuals who exhibit symptoms of testosterone deficiency (reduced libido, gynecomastia, depression, low bone mineral density, decreased energy, low muscle mass/strength and poor cardiovascular health profile) and low testosterone serum levels (< 12 nmol/l) [78, 79]. The risks associated with TRT include infertility (in young), cardiovascular disease and prostate cancer and therefore, should be assessed on a case by case base by a clinician [79, 80]. It has also been reported that an important motivation to take AAS was to “slow the ageing process” in older males, which may exhibit greater health risks compared to clinically prescribed TRT [81].

In summary, chronic AAS use increases skeletal muscle anabolism, which results in skeletal muscle hypertrophy, improved function and body composition via genomic, non-genomic and anti-catabolic signalling pathways. Nevertheless, the use of AAS has negative consequences on overall metabolic health through altered lipid metabolism and therefore an increase in CVD risk. With CVD being the number one cause of deaths globally, the potential clinical benefits of AAS use on skeletal muscle are far outweighed by the negative outcomes on cardiovascular health [31].

## Impact on metabolic health

### Lipid metabolism

**Fig. 3:**
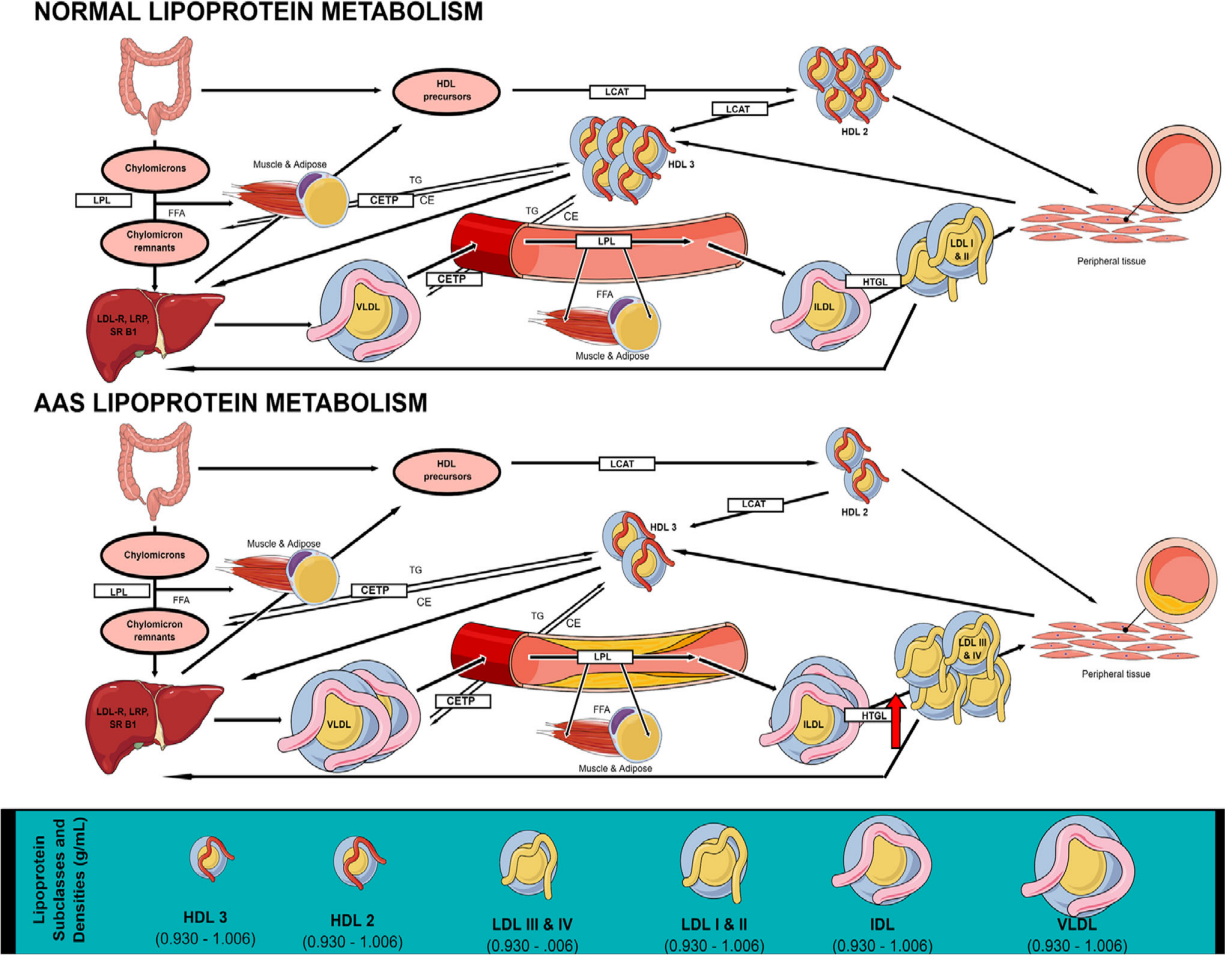
Normal and AAS-influenced lipoprotein metabolism. During normal lipoprotein metabolism, intestinally produced chylomicrons carrying dietary lipids are hydrolysed by lipoprotein lipase (LPL). FFA are liberated and taken up by the liver, muscle and adipose tissue. Resulting chylomicron remnants are taken up by the liver via low-density lipoprotein receptor (LDL-R) and the LDL receptor-related protein (LRP). Meanwhile, hepatically produced VLDL transport cholesterol esters (CE) and TG through blood vessels, during which they undergo hydrolysis, releasing FFA which are taken up by peripheral tissues. This loss of TG means VLDL particles decrease in size (and therefore density) and become cholesterol-enriched and known as idLDL. Due to the action of HGTL, IDL particles become even smaller and known as LDL. LDL particles have an increased propensity to deposit cholesterol in peripheral tissues; however, they primarily transport cholesterol to the liver, where they are taken up by the LDL-R. The intestine also produces precursors which contribute towards the production of HDL. Small HDL3 particles acquire CE and TG and form larger HDL2 particles which, with the assistance of lecithin–cholesterol acyltransferase (LCAT), subsequently exchange CE for even more TG with VLDL particles and chylomicrons, before travelling to the liver where they are taken up by scavenger receptor B1 (SR-B1) or LDL-R. During AAS-influenced lipoprotein metabolism HGTL is upregulated, resulting in a preponderance of more atherogenic small, dense LDL III and IV particles, as opposed the larger and more buoyant LDL I and II particles found in normal lipoprotein metabolism. There is also a severe decrease in the number of HDL 2 and 3 particles overall, which are generally regarded as being atheroprotective.

Dyslipidaemia is associated with an increased CVD risk and is underpinned by high levels of triglycerides > 150 mg/dL, LDL-C > 116 mg/dL and/or low levels of HDL-C < 40 mg/dL in males and < 50 mg/dL in females [82, 83]. Although only triglycerides and HDL-C are considered components of MetS, sdLDL-C is considered an additional element to this disease [30]. LDL can be separated into 4 groups: large and buoyant (lbLDL), intermediate size and density (idLDL), small and dense (sdLDL) and very small and dense (vsdLDL) [84]. High circulating particles of sdLDL and vsdLDL indicate a greater risk of CVD events compared to total LDL alone [84, 85]. Cholesterol is primarily synthesized in the liver and circulates around the body as very-low-density lipoprotein (VLDL) (rich in TG) of which apolipoprotein B100 (ApoB) is the major apolipoprotein (Fig. 3) [86]. Upon interaction with lipases at various tissues, the VLDL containing TG are hydrolysed, and free fatty acids are released for energy or subsequent storage as adipose tissue [87]. The remaining lipoprotein is now cholesterol-rich, TG poor LDL (or LDL-C). This LDL will bind to the hepatic LDL receptor to increase LDL-C clearance [87]. With dysregulated metabolism, as observed in MetS, there is an increase in circulating sdLDL which, has a lower affinity for the LDL receptor, therefore, having a reduced clearance rate, subsequently increasing circulating levels and CVD risk [88]. The sdLDL can also penetrate the arterial wall easier compared to lbLDL due to its small size increasing the risk of trapping ApoB depositing atherogenic cholesterol and increasing the risk of a CVD event [89]. High-density lipoprotein (HDL), particularly subfraction HDL_2_ transports cholesterol away from peripheral tissue, including arterial lesions, to the liver to be excreted, through a process of reverse cholesterol transport, thereby reducing CVD risk [90, 91]. HDL of which apolipoprotein A1 (ApoA1) is the major apolipoprotein, also has an anti-inflammatory and antioxidant effect on the vascular system further reducing the potential of CVD [92]. Use of AAS has shown a reduction in HDL-C of ≥ 70% and increased LDL-C levels of > 20% [33]. Testosterone has been reported to significantly decrease HDL-C, although with differential dose and time responses. Increasing doses of Test E for 20 weeks in resistance-trained males has been reported to have an inverse dose-response relationship with HDL-C and Apo A1 but only 600 mg/wk was significantly (*P* < 0.001) different to baseline levels [93]. However, in contrast, 150 mg/wk for 2 weeks and a 300 mg dose of testosterone cypionate on week 3 resulted in the largest decrease in HDL-C but no further decrease was observed with 600 mg/wk for a further 4 weeks [94]. Furthermore, 3 weeks of 600 mg/wk Test E administration in inactive ageing males resulted in significant decreases in HDL-C, particularly HDL_2_ [95]. Although 200 mg/wk of Test E in resistance training males showed significant decreases in HDL-C after 6 weeks, no effect was observed on HDL_2_ [96]. In healthy males, 200 mg/wk of Test E administration for 12 months had dramatic significant (*mean*:1.15 mmol/L to 0.09 mmol/L, *P* < 0.05) decrease in fasting HDL-C levels. Interestingly, neither study observed significant deleterious changes in LDL-C or TG levels in fact, Thompson et al. (1989) reported a significant (*P* < 0.05) decrease in LDL-C.

Nandrolone administration has reported contrasting effects on lipid metabolism. HDL-C has been reported to significantly decrease after a 200 mg starting dose of nandrolone and a further 100 mg/wk for a total of 8 weeks in male bodybuilders [97]. Although in a similar design and population, 200 mg/wk of nandrolone for 8 weeks resulted in no significant change in HDL-C [98]. In healthy adults, 100 mg/wk for 6 weeks resulted in no change in HDL-C [99]. No effect was observed on LDL-C, TGs, Apo A1 or Apo B levels in the above studies [97–99]. Nandrolone administration (200 mg/wk) for 6 months in ageing males undergoing haemodialysis resulted in significantly reduced HDL-C and increased apo B levels but had no effect on TG or Apo A1. [100]. In post-menopausal women, 50 mg/wk of nandrolone for 3 weeks significantly decreased HDL-C and Apo A1 levels [101]. In male bodybuilders, 42 mg/wk of oral stanozolol administration has shown to significantly reduce HDL-C, ApoA1 and TGs after 6 weeks while also increasing LDL-C [96]. In healthy males, one intramuscular injection of 50 mg of stanozolol resulted in a significant reduction and increase in HDL-C and LDL-C levels respectively 28 days later [102]. Both returned to baseline levels after 56 days [102]. Similar results have been observed in postmenopausal females with osteoporosis, as 42 mg/wk of oral stanozolol resulted in significant reductions in HDL-C and ApoA1 levels after 2 weeks and was maintained until the end of the treatment at 6 weeks along with an increase in LDL-C levels [103]. No change was observed in TG levels [103].

Differences in study designs, populations and lack of dietary control in some studies has resulted in differing responses in lipid metabolism with AAS administration. Nonetheless, increasing doses of testosterone administration has a large negative impact on HDL-C with no adverse effect on other lipid markers. Although inconsistent, the negative effects of nandrolone administration are primarily observed on HDL-C levels, however, nandrolone does appear to consistently reduce lipoprotein(a) (Lp (a)) levels [98, 100, 101], an independent risk factor of CVD [104, 105], yet further research is warranted on the potential benefits of nandrolone, if any. In contrast, stanozolol administration may have a greater deleterious effect on lipid metabolism as it has shown to negatively affect LDL-C and HDL-C levels.

Individuals who use AAS for appearance and performance reasons typically do not use one type of AAS but rather administer a polypharmacy regime which may lead to different implications on lipid metabolism.

Early studies reported that after 8 weeks of AAS administration, HDL-C and LDL-C significantly (*P* < 0.01) decreased by 49% and increased by 31% respectively [106]. Similarly, this suppression on HDL-C, particularly HDL_2,_ is maintained after 14 weeks of self-administration [98]. However, Bonetti et al.. only reported a significant (*P* < 0.05) decrease in HDL-C after 18 months [107]. The method of using self-administrating participants results in a variety of AAS dosages, types and cycles being used which may lead to different health outcomes thereby making comparisons between studies difficult. Critically, however, although they may be less controlled, they may be more representative of the population compared to randomised controlled trials as it replicates the AAS and AAS-related polysubstance methods used by this unique population. A more recent cross-sectional study reported similar results in which current users of AAS, displayed 45% lower HDL-C, and 26% and 35% higher LDL-C and TG levels vs. non-AAS using controls (all *P* < 0.01) [37]. A case study of prolonged AAS use in a 35-year-old male demonstrated an almost 100% decrease in HDL-C and a 100% increase in LDL-C during 5 years of AAS cycling [108]. Similarly, in females, HDL-C is shown to be significantly depressed with chronic AAS use compared to healthy controls. While AAS use may also exhibit an increase in plasma TG, data remains equivocal as this was only reported by Moffatt et al. [109–111]. In addition to small sample sizes in female studies, the variety in AAS use, type, dose and frequency might explain the differences in results. Although, the lipoprotein profile is undoubtedly impacted by chronic AAS use and therefore highlights the increased risk of future CVD incidence, due to the uncontrolled self-administration of AAS and other anabolic substances the severity in which it impacts health can be variable. Conversely, AAS polypharmacy is also reported to improve Lp (a) levels, similar to the effect of nandrolone administration alone [98]. Self-administration of a variety of AAS resulted in a significant (*P* < 0.05) decrease in Lp (a) after 8 weeks and was maintained after 14 weeks [98]. However, 24 months of AAS use did not result in a significant decrease (mean ± SD; 179 ± 117 vs. 137 ± 80 mg/dL, *P* > 0.05) in Lp (a) [107]. Although non-significant, it may be clinically significant as Lp(a) levels > 180 mg/dL are considered high risk of CVD [82]. The implications of AAS induced improvements in Lp(a) concerning CVD risk are unclear and warrant further investigation. Use of AAS also doesn’t appear to negatively impact TG levels in males as only one cross-sectional study reported significantly (*P* < 0.01) higher TGs (although not clinically significant < 1.7 mmol/L) with AAS use, yet self-administration studies showed no significant negative effect [98, 106, 107]. The mechanisms by which AAS negatively impact lipid metabolism are not fully understood, but the upregulated activity of hepatic triglyceride lipase (HTGL) has been implicated (Fig. 3) [95, 96]. Phospholipase activity of HTGL catabolises HDL-C and its removal from the plasma and conversion of idLDL to sdLDL [42, 112]. Research of the impact of AAS use on LDL density are limited with most focusing on total LDL-C however, one randomised controlled trial investigated the short-term (3 weeks) effects of TestE administration on cholesterol associated with LDL density by density gradient ultracentrifugation (DGUC) [95]. In older eugonadal males (mean 71 years old), 600 mg of TestE increased sdLDL-C indicating an increase in CVD risk [85, 95]. Unpublished data, by the authors, showed no significant (*P* > 0.05) difference in a cross-section of AAS using males and healthy controls in sdLDL-C. Further research is warranted on the effect of AAS use on LDL density and its associated CVD risk. The type of AAS and route of administration also has an impact on the effect of HTGL activity and lipoprotein levels. Orally administered stanozolol showed a significant (*P* < 0.05) increase in HTGL activity, leading to a significant (*P* < 0.05) increase and decrease in LDL-C and HDL_2_-C respectively whereas injected TestE showed no significant (*P* > 0.05) change in HDL_2_-C after 6 weeks, but a significant (*P* < 0.05) decrease in LDL-C [96]. The slower liver clearance rate of orally administered AAS compared to injected AAS could have a greater detrimental effect on metabolic health and also increase the risk of hepatoxicity [113–115]. Interestingly, the effect of AAS on the lipoprotein profile is reversible, as former users of AAS with long term discontinuation of at least one year, are reported to have healthy lipoprotein levels [37, 116]. The reversible effects may be seen as early as 10 weeks of AAS cessation as shown by a case study in a 35-year-old male [108].

### Glucose metabolism and VAT

Key features associated with MetS are IR and VAT [30]. IR is the precursor of the development of Type 2 diabetes (T2D), with lipid accumulation and inflammation being implicated as the primary triggers [117–119]. IR can be measured by the hyperinsulinemic-euglycemic clamp with IR being defined as a glucose disposal rate below 5.6 mg/kgFFM + 17.7/min [120]. Skeletal muscle is the largest tissue for insulin-induced glucose uptake [121]. Insulin binds to the insulin receptor on the cell membrane causing its tyrosine phosphorylation of the receptor (Fig. 1). The now activated insulin receptor causes phosphorylation of insulin receptor substrate-1 (IRS-1) on tyrosine residues, which allows the recruitment of the Type IA phosphatidylinositol 3’ kinase (PI3K). PI3K catalyses the formation of PI(4,5)-bisphosphate to PI(3,4,5)-trisphosphate thus recruiting 3’ phosphoinositide-dependent kinase-1 (PDK-1). PDK-1 phosphorylates protein kinase B (PKB) (also known as Akt) and the atypical protein kinase C (PKC) [118, 119, 122, 123]. Akt phosphorylates 160-kDa substrate of Akt (AS160) which stimulates translocation of GLUT4 storage vesicles to fuse at the cell surface to release GLUT4 into the plasma membrane allowing cellular glucose uptake [124, 125]. However, within IR tissue this signalling cascade is diminished possibly due to increased circulating fatty acids, inflammatory cytokines and/or reactive oxygen species (ROS) which result in serine/threonine phosphorylation of IRS-1. This reduces Akt activity and glucose uptake and negatively affects other downstream signalling such as protein synthesis and apoptosis (Fig. 1) [119, 122, 126]. Acute testosterone administration has shown to activate the PI3K/Akt pathway and GLUT4 translocation *in vitro* indicating an increase in cellular glucose uptake [54]. However, supraphysiological levels of testosterone and nandrolone have been reported to significantly (*P* < 0.05) diminish the response of insulin-induced glucose uptake in rodents [127, 128]. Rodents also showed impairments in gluconeogenesis, most likely due to the high fasting insulin levels [128]. In contrast, increasing doses of testosterone (25–600 mg/wk) for 20 weeks had no significant effect on insulin sensitivity in resistance-trained males [93]. Additionally, in a double-blind crossover design, 300 mg/wk of Test E and nandrolone administration for 6 weeks did not affect glucose tolerance or fasting insulin levels in healthy males [129]. Although research is lacking, females who use AAS for performance are reported to display reduced insulin sensitivity [130]. In healthy females, up to 12 days of methyltestosterone dosing (5 mg), showed a significant (*P* < 0.05) reduction in whole-body insulin sensitivity [130]. Similarly, in postmenopausal females, 120 mg of testosterone undecanoate per week resulted in a significant decrease in insulin sensitivity [131]. Hyperandrogenism in females is a significant risk factor in developing polycystic ovary syndrome (PCOS) and PCOS increases the risk of developing MetS although the risk of developing CVD is currently unclear [132–135]. Interestingly, muscle strength determined by bench press and handgrip test was shown to be significantly (*P* < 0.05) higher in females with PCOS compared to healthy controls, further indicating that hyperandrogenism may be implicated in PCOS and MetS [136].

Although individual AAS use may not result in reduced insulin sensitivity in males [93, 129], limited research suggests chronic AAS polysubstance use may be detrimental to glucose metabolism as shown by Cohen et al. [137]. Powerlifting steroid users (PS) were shown to have similar fasting glucose levels as non-using powerlifters (NP) and sedentary participants; however, they had significantly (*P* < 0.05) higher fasting insulin levels that were similar to those observed in obese participants [137]. An oral glucose tolerance test (OGTT) also revealed the PS to have a significant (*P* < 0.05) 2-fold increase in post-glucose glycaemia compared to NP, which was a similar increase to the obese group. Post-glucose insulinaemia in the PS group was also significantly (*P* < 0.01) higher compared to all groups, with it being at least 2-fold higher compared to obese participants [137]. The authors only report participants use of AAS although insulin is commonly used for its anabolic potential and may have also been used by participants which may have impacted the results. More recently in males, an OGTT between healthy controls, steroid-using bodybuilders and former steroid-using bodybuilders (mean discontinuation of 2.5 years) revealed that current and former AAS users had significantly (*P* < 0.05) impaired glucose tolerance compared to healthy controls [37]. Reduced insulin sensitivity in former AAS users, was associated with higher % body fat, which may be due to reduced testosterone levels compared to healthy controls [37, 138].

Chronic AAS use suppresses the hypothalamic-pituitary-testicular (HPT) axis resulting in reduced endogenous testosterone production [39]. Low testosterone levels reduce insulin sensitivity and increases risk of developing MetS and CVD [139]. Interestingly, although current users of AAS had significantly (*P* < 0.001) lower % body fat compared to healthy controls and former users, they had significantly (*P* < 0.05) greater levels of VAT and reduced adiponectin and leptin levels which are all independent predictors of IR, T2D and MetS [37, 140–143].

However, a randomised controlled trial of the dose-response of TestE for 20 weeks showed significant (*P* < 0.05) decreases in VAT with higher doses, indicating supraphysiological doses do not increase VAT [144]. Although cross-sectional studies cannot determine causation, it may be more representative of long term AAS and AAS-associated polysubstance use in this case. Individuals typically use a range of AAS types and other complementary drugs; doses, cycles, methods of administration and for years rather than weeks or one AAS which may explain the differences in results.

**Fig. 4:**
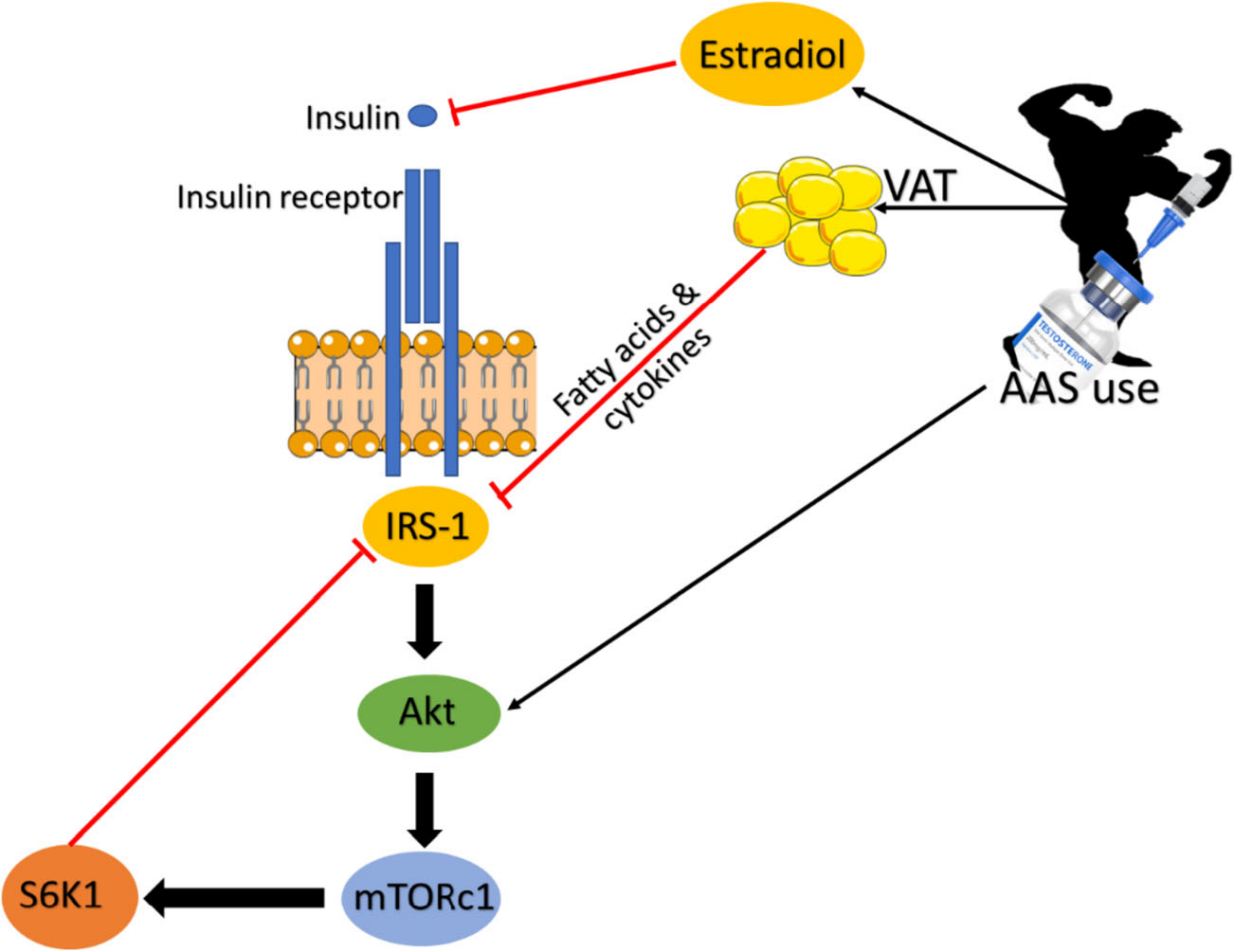
Potential mechanisms of insulin resistance with chronic anabolic steroid use. Chronic upregulation of S6K1 via activation of PI3K/Akt signalling cascade by AAS may reduce insulin sensitivity due to inhibition of IRS-1 by S6K1 as seen with nutrient overload models. Furthermore, chronic AAS use may lead to an increase in VAT increasing circulating fatty acids and/or inflammatory cytokines causing inhibition of IRS-1 and reducing insulin sensitivity. Aromatisation of testosterone may lead to increasing levels of Estradiol causing IR by binding to insulin and the insulin receptor.

Accumulation of VAT is an important indicator of glucose tolerance, MetS and CVD risk, much more so than subcutaneous adipose tissue (SAT) [143, 145, 146]. In healthy adipose tissue, when surplus energy is consumed, the energy is stored in SAT. However, in unhealthy or IR adipose tissue, the excess energy will be deposited in VAT and a variety of organs including muscle tissue [147, 148]. The lipolytic rate of VAT is increased compared to SAT due to the increased effect of pro-lipolytic catecholamines and decreased effect of anti-lipolytic insulin. This increases the flux of FFA to the liver, which may further increase hepatic IR [146]. Though, with AAS use, VAT was associated with lower lipolysis rates as determined by lower levels of plasma glycerol [37]. The unusual lower lipolytic activity may be attributable to reduced activity of catecholamines due to AAS compounds such as nandrolone downregulating β3-adrenoceptor expression [149]. In addition to being involved in lipid storage and mobilisation, adipocytes are also an endocrine tissue, releasing cytokines and adipokines. An increase in VAT leads to a pro-inflammatory state as shown by an increase in C-reactive protein (CRP) and tumour necrosis factor-alpha (TNF-a) which may further increase IR [150, 151]. As skeletal muscle is the largest tissue for glucose disposal, increases in muscle mass should improve insulin sensitivity; paradoxically, these results indicate that chronic AAS may cause tissue IR. This may be due to an imbalance of regulatory adipokines and cytokines from increased VAT levels and circulating lipids leading to a decreased/delayed stimulus of the PI3K/Akt signalling cascade in response to glucose ingestion, as also observed in T2D individuals [119]. This dysregulated metabolism leads to a continuous cycle of VAT and IR that potentiate each other. Furthermore, nutrient overload is reported to increase IR via mTORc1 dependent pathway. Chronic activation of S6K1 mediated by mTORc1, inflicts serine phosphorylation of IRS1 leading to reduced insulin sensitivity [152, 153]. For example, chronic high glucose concentrations in murine skeletal muscle cells (C2C12 myoblasts) induce IR and reduced Akt stimulation; however, inhibition of mTOR/S6K1 signalling with rapamycin restored insulin induced Akt stimulation [154]. It may be possible that chronic AAS use, leading to hyperactivation of mTORc1/S6K1 signalling may cause IR (Fig. 4). Estradiol has shown to be significantly (*P* < 0.01) higher with AAS use compared to healthy controls and may also be a cause of IR in this population. The conversion of testosterone to estradiol resulting in a decrease in the testosterone to estradiol ratio has been implicated in the development of MetS in older males [155]. Additionally, estradiol is reported to bind to insulin and the insulin receptor further highlighting its potential role in inducing IR (Fig. 4) [156].

Research is currently lacking on the prevalence of increased levels of VAT with AAS use, most likely due to AAS typically reducing fat and to its dysregulation of insulin sensitivity. This field of research warrants further investigation.

### Hypertension

Hypertension is highly associated with MetS and CVD risk [30, 157]. Hypertension is caused by an increase in vascular stiffness due to degenerative changes in the extracellular matrix (ECM) derived from an imbalance of arterial scaffolding proteins such as elastin and collagen [158]. Chronic low-grade inflammation, underpinned by cytokines such as CRP, TNF-a and interleukin-6, induced by ageing, T2D or an atherogenic lipid profile results in endothelial and smooth muscle cell proliferation, hypertrophy, remodelling and apoptosis [159, 160]. This vascular proinflammatory state characterised by angiotensin II results in upregulation of matrix metalloproteinases (MMPs) leading to degradation of elastin fibres and increased collagen deposition resulting in ECM remodelling and arterial stiffening [161, 162]. Furthermore, metabolic disorders such as T2D can cause disruption of vasodilation (nitric oxide) and vasoconstriction (endothelin) regulators resulting in hypertension [160].

Hypertension, as a result of chronic AAS use, is under debate due to conflicting data [33]. Although further research is required, there is some evidence to suggest that chronic AAS use in males may lead to increased BP [33, 163–165]. By contrast, early studies suggested that AAS use did not have a detrimental effect on BP even with 24-hour monitoring [97, 106, 166]. Short-term (< 8 weeks) testosterone (200 mg/wk) and nandrolone (100–200 mg/wk) administration resulted in no change in systolic or diastolic BP [97, 167] however, long term-controlled studies are lacking.

Lenders et al., reported AAS polysubstance use to have a significant (*P* < 0.05) increase in systolic BP (SBP) after an average AAS use of 5 months although the increase was not clinically relevant (118 ± 2.2 to 121 ± 2.4 mmHg) [106]. Nevertheless, more recent studies have shown chronic AAS polysubstance use to have significantly (*P* < 0.05) higher SBP compared to healthy controls and former AAS users [163, 165]. These results may be clinically relevant as mean SBP was reported to be 132 mmHg and 138 mmHg in current AAS users [163, 165]. Current and former AAS users were also reported to have significantly (*P* < 0.05) increased aortic stiffness. These higher levels in aortic stiffness and SBP were associated with the significantly (*P* < 0.05) lower mid-regional pro-atrial natriuretic peptide (MR-proANP) in AAS users [163]. ANPs regulate vasodilation, reduce renin-angiotensin-aldosterone system activity and sympathetic nerve activity; yet, high levels of MR-proANP are associated with hypertension and incidence of mortality [168, 169]. The conflicting results regarding hypertension with AAS use may be partly due to differences in study designs. Repeated measure designs as implemented in the early studies are more indicative of causal effects compared to the most recent cross-sectional studies; however, the cross-sectional studies have larger sample sizes and potentially greater power but only association can be conferred. The lack of control on AAS type and quantity also makes it difficult to compare findings. Nonetheless, chronic AAS use may have detrimental effects on the vasculature and consequently causing hypertension and increased risk of CVD, but more long-term controlled studies are required.

## Reducing CVD risk

In addition to increased LDL-C, research indicates that AAS users may develop MetS due to having low HDL-C, IR, possible hypertension and increased VAT. Considering this, they share a similar metabolic phenotype to sedentary/obese populations and have an increased risk of CVD incidence [30, 32, 33, 37, 98]. As MetS is typically associated with obese/sedentary populations, treatments include inducing weight loss through improving lifestyle behaviours (exercise and nutrition) and bariatric surgery or pharmaceutical medication to alter negative metabolic function [170]. However, these interventions would not apply to AAS users due to their already low body fat and high physical activity [170].

To reduce MetS and CVD risk, cessation of AAS use is highly recommended as it has been shown to at least improve the lipoprotein profile, yet may have lasting effects on insulin sensitivity, BP and VAT levels [37, 106, 108, 163]. Unfortunately, total cessation can lead to withdrawal symptoms such as hypogonadism in males, infertility and depression [7, 39]. Suppression of the HPT axis results in low endogenous testosterone production leading to decreased sexual function, such as erectile dysfunction and reduced libido and may be dependent on the dose and duration of AAS use [171]. These symptoms promote relapse and AAS dependency and must be treated accordingly with pharmaceutical and cognitive behaviour therapies to help with AAS cessation and prevent relapse [172]. AAS use may also lead to gynecomastia, due to an increase in the estrogen to testosterone ratio via an increase in aromatase activity resulting in the conversion of testosterone to estradiol [173]. Therefore, use of estrogen receptor antagonists are typically used in conjunction with AAS, particularly during times of AAS cessation [41, 173]. On cessation of AAS, pharmaceuticals such as hCG, aromatase inhibitors and SERMs can reduce withdrawal symptoms although current evidence is lacking on its benefits [7, 41, 174]. Hypogonadism is also associated with MetS, therefore, total cessation of AAS use may not improve MetS symptoms and CVD risk [175, 176]. It may be feasible to decrease AAS use instead of total cessation as low testosterone (40–80 mg per day) treatment in males with hypogonadism has been shown to improve MetS markers [177]. However, further clinical trials in AAS users, who wish to stop, are required before any true treatment can be recommended. Additionally, many users of AAS rely on information from websites or online forums for post-cycle therapy which may lead to mismanagement of AAS withdrawal symptoms [178]. It’s imperative that users of AAS seek professional advice while it is also equally important that clinicians aim to help in a non-judgemental way to reduce the likelihood of permanent adverse effects of AAS use. There is currently a lack of research on the effects of AAS withdrawal in females although they are likely to improve their lipoprotein profile but may also require treatment for depressive symptoms and amenorrhea [179–181].

## Conclusion

CVD is the number one cause of deaths globally, with the obesity epidemic being a major contributor. The increasing prevalence of AAS use, particularly in young males, will exacerbate the current CVD rates. Chronic use of AAS leads to increased skeletal muscle hypertrophy and improved performance by binding to the AR. Activation of the AR by AAS leads to enhanced gene transcription, second messenger signalling, and satellite cell activation leading to increased muscle protein accretion and synthesis and possibly decreased catabolism. However, chronic AAS use not only leads to impaired cardiac function but also MetS and associated dysregulated metabolic health (IR, dyslipidaemia, VAT and BP) which is more commonly related with the sedentary/obese population. Effective management of AAS and AAS-related polypharmacy use in the first place, together with appropriate guidance on AAS cessation is key, both of which may be managed by education and psychological interventions to ultimately improve health. Therefore, further research is warranted on the long-term effects of AAS use and cessation on markers of metabolic health to provide accurate information on the potential harms in males and females. Further research is also required for treatments to aid AAS cessation and combat adverse metabolic health in this population.

## Data Availability

Not applicable.
